# Solar Energy and Solar Cells

**DOI:** 10.3390/nano11102682

**Published:** 2021-10-12

**Authors:** Dongling Ma

**Affiliations:** INRS-Energy, Materials and Telecommunications, Varennes, QC J3X 1S2, Canada; Dongling.Ma@inrs.ca

Thanks to the helpful discussions and strong support provided by the Publisher and Editorial Staff of *Nanomaterials*, I was appointed as a section Editor-in-Chief of the newly launched section “Solar Energy and Solar Cells” earlier this year (2021). I am honored to accept this position and to be part of the editorial team, and I look forward to contributing to the development of this extremely valuable section and to the wider nanomaterials community.

The year of 2021 is destined to be recorded as an extraordinary year. Responding to the seemingly never-ceasing, elusive fluctuation of the global COVID pandemic that has caused huge waves of losses to human life, public health, and the economy, we are fundamentally changing our way of living, working, and socializing. Despite suffering from the initial shock caused by the outbreak in 2021, we rapidly recovered from being helpless and started to take necessary actions and measures, including advancing effective vaccines. Can we do the same for Earth, whose well-being is currently endangered? Earth is becoming extremely sensitive to even a small increase in greenhouse gases (such as CO_2_) in the atmosphere, and, therefore, we are facing tremendous and persistent challenges of climate change and extreme weather. In 2021 alone, we witnessed unusual killer floods in China and Germany, “scary” heat in the pacific northwest of the USA and Canada, and devastating wildfire [1], just to name a few. The good news is that an increasing number of countries, including, for example, Canada, France, Germany, Italy, Japan, the United Kingdom, and the United States of America (USA), are committed to realizing net-zero emissions by 2050 [2]. A huge leap in harvesting, using, and deploying solar energy is key to successfully accomplishing this formidable task and requires significant, joint forces and coherent actions. ***The launching of this new section timely echoes this call***.

The section “Solar Energy and Solar Cells” publishes original high-quality research, communications, articles, and review articles in the broad areas of ***solar-related materials, devices, processes, and technology, as well as simulations, computational studies, and emerging machine learning*** applied to these research topics. It covers important research topics, such as ***solar cells***, ***solar fuel***, ***solar energy storage,*** and ***lifecycle analysis*** of solar-related materials and technology.

To achieve the goal of publishing high-quality, high-impact research in the areas of solar energy and solar cells, we have assembled an excellent editorial team, composed of 10 world-leading scientists: Prof. Guozhong Cao (University of Washington, Seattle, WA, USA), Prof. Guanying Chen (Harbin Institute of Technology, Harbin, China), Prof. Yuning Li (University of Waterloo, Waterloo, ON, Canada), Prof. Hong Liu (Shandong University, Jinan, China), Prof. Quinn Qiao (Syracuse University, Syracuse, NY, USA), Dr. Aurora Rizzo (National Research Council, Rome, Italy), Prof. Baolian Su (University of Namur, Namur, Belgium), Prof. Baoquan Sun (Soochow University, Suzhou, China), Prof. Yang Yang (University of Central Florida, Orlando, FL, USA), and Prof. William Yu (Louisiana State University in Shreveport, Shreveport, LA, USA).

On behalf of the section editorial team, I am delighted to invite you to submit your original work and review papers. I also look forward to your suggestions to help improve this section. “No Effort Is Too Small” [3].
